# The Homeless Poor of London

**Published:** 1905-03-18

**Authors:** 


### The Hospital.

A Journal of

The Medical Sciences and Hospital Administration.

Vol. XXXVII.—No. 964 MARCH 18, 1905.

### The Homeless Poor of London.

The return published by the Medical Officer of Health for the County of London, showing the number of persons who were found wandering and homeless, or sleeping on staircases and in doorways in the metropolis, on the night of February 17th, is a document suggestive of many considerations. The figures were obtained by means of a careful inquiry instituted under the direction of the London County Council, and the inspectors employed in collecting them were materially assisted by the experience gained at a similar but less complete census which was made in January 1904. The result of their labours was to discover 1,869 men and 312 women in the streets, or on staircases or under arches, but apparently no children. Of the total number, 827 men and 39 women were in Wych Street, Strand, and 161 men and 60 women were in Whitechapel Road, both localities in which food was being given away by the Salvation Army. In a tent of the Church Army in Clare Market, Strand, 300 men were, or had been, chopping wood. These men had received two meals during the night, and were given tickets for beds in common lodging-houses, but these tickets were not available until the following night, so that the 300 men should be added to the rest, thus raising the number to 2,481. On the same night there was vacant accommodation in the common lodging-houses for 4,610 men, 856 women, and 62 couples; and there was vacant accommodation in the casual wards for 337 men, 213 women, and 117 women and children; or, on the whole, for about two-and-a-half times the amount of the actual vagrancy which was discovered. It follows that the whole of the women found wandering could have been accommodated in casual wards; and, considering that many of them were probably too tired and too hopeless to undertake a journey on a mere chance of being received, it seems to be a matter for regret that the authorities of the charitable organisations mentioned had not placed themselves in telephonic communication with the wards, and so rendered themselves able to say to every woman concerned, and to as many men as possible, that at such or such a place they would find shelter and food. In the case of the men, it must be assumed that at least a large proportion of them were not in possession of the amount of money required in order to obtain a bed at a common lodging-house; but it is at least probable that some of them were kept in the streets by the curious instinct of vagrancy which in some seems to be either a survival or an example of atavism, and to be derived from other times and other manners than our own. Whatever numerical allowance should be made for such possibilities, the fact remains that the night's tale of vagrancy is an appalling one, and that the vagrants themselves constitute a dark blot upon our civilisation. In the aggregate they are a serious source of disease and danger to the public.

It is, moreover, tolerably manifest to all who have bestowed real consideration upon the questions involved, that vagrancy and homelessness are not to be dealt with by what is called (often very deceptively) charity, even when that is directed by large and wealthy organisations. These do not touch the roots of the evil, and the evil is an increasing one. The number of homeless persons discovered last month was greater by 684 than the number discovered by a similar inquiry last year; and will probably be again exceeded in 1906. The only proper view of the case would be to regard vagrancy in itself not as a claim upon the benevolent, but as an offence punishable by law, and only capable of being excused on very valid grounds. The organisation for dealing with distress which Mr. Long has originated will, we trust, not be suffered to decay as a consequence of his removal from the Local Government Board to the Irish Office, and the penalisation of vagrancy, which already obtains in countries where (probably on that very account) it is much less common and less obnoxious than with us, should be at least one of the aims to which this organisation will be directed. It is necessary to admit that even an industriously-disposed and honest man may be so broken down by adverse circumstances, by illness, or by want of employment, as temporarily to lose heart and strength and to let himself go downhill with a sort of despairing indifference; but the true character of such a man would never be concealed from any who had acquired experience in dealing with cases of distress, and a hand stretched out in timely help would suffice, as a rule, to restore him to his original level. Admitting his existence, he is still one of a small minority. The real difficulty of the problem is how to deal with the numbers who never did a day's honest work in their lives, and who never intend to do one, who take to vagrancy as to their natural condition, and who, if they think at all, balance its license and its occasional prizes against sufferings and discomforts which would be intolerable to persons differently constituted, but which they have come to regard very much as one of the mere chances of their war against industry and against society. For such people the strong arm of the law should be exerted without hesitation, and the mere fact of their admitted incapacity to maintain themselves in decency and comfort should be regarded as a valid reason for placing them more or less permanently under the control of a taskmaster. For them the labour colony should be a distinctly penal settlement, in which the sexes should be separated, and from which any children belonging to the delinquents should be removed. Such colonies could be maintained at far less cost to the nation than the amount which is now expended in misdirected almsgiving to worthless and hopeless subjects. It is too much, of course, to expect any useful social legislation from the present Parliament, which has lately been affording only too much evidence of the indifference to their duties of members who will not seek re-election, and of the subservience to agitators of those who will. Whenever we are so fortunate as to obtain a strong Government, with a safe majority in a new House of Commons, we may hope that the questions of poverty and of vagrancy will receive early and grave consideration.

